# Severe Stroke Patients With Left-Sided Occlusion of the Proximal Anterior Circulation Benefit More From Thrombectomy

**DOI:** 10.3389/fneur.2019.00551

**Published:** 2019-05-28

**Authors:** Zibao Li, Zhaohu Chu, Shoucai Zhao, Lingsong Ma, Qian Yang, Xianjun Huang, Zhiming Zhou

**Affiliations:** Department of Neurology, Yijishan Hospital, Wannan Medical College, Wuhu, China

**Keywords:** stroke, sides of occlusion, intervention, stent, thrombectomy

## Abstract

**Background and Purpose:** Endovascular thrombectomy improves the functional independence of patients with proximal anterior circulation occlusion. However, a subset of patients fail to benefit from thrombectomy procedures, the reasons for which remain poorly defined. In this study, we investigated whether the effectiveness of thrombectomy was affected by left- or right-sided occlusion among patients with similar stroke severities.

**Methods:** Patients with proximal anterior circulation occlusion (internal carotid or M1 of middle cerebral artery) treated with the Solitaire stent retriever within 8 h of the onset of acute ischemic stroke were enrolled from the Yijishan Hospital of Wannan Medical College. Stroke severity was measured using the National Institutes of Health Stroke Scale (NIHSS) on admission. The functional outcomes were assessed using the modified Rankin scale (mRS) at 90 days.

**Results:** We enrolled 174 patients including 90 left-sided occlusion and 84 right-sided occlusion. The NIHSS scores on admission were higher in the left-sided (median, 19; interquartile range, 16 to 20) compared to the right-sided occlusion group (median, 15, interquartile range, 13 to 18) (*P* < 0.001). Following adjustment for potential risk factors, patients with left-sided occlusion had higher rates of functional independence (mRS ≤ 2) and lower rates of mortality (mRS = 6) compared to the right-sided occlusion patients (39.5 vs. 19.6% and 28.9 vs. 47.8%, respectively) in the severe stroke group (NIHSS ≥ 15).

**Conclusions:** In severe stroke patients with proximal anterior circulation occlusion, stent retriever thrombectomy within 8 h of the onset of symptoms provides more benefits to left-sided occlusion.

## Introduction

Endovascular thrombectomy can effectively treat acute ischemic strokes in patients with occlusion of proximal anterior circulation (1–5). Subsequent two randomized controlled trials (RCTs) that were performed using imaged-based screening to extend the time window of endovascular therapy (6, 7), suggested that neuroimaging evaluations improved the prediction of individualized responses to reperfusion therapy. However, these RCTs resulted in a 29–67% poor outcome with 9–25% mortality (1–7), indicating that a significant number of patients might not benefit from endovascular thrombectomy. The identification of predictors affecting the benefit of thrombectomy would hold great value for stroke therapies.

Left and right hemispheres have similar anatomical structures, but differ in terms of neurological function. More than 95% of right-handed individuals and 70% of left-handed individuals had the left hemisphere (typically dominant hemisphere) for language function, while the right hemisphere (typically non-dominant hemisphere) is specialized for emotional and non-verbal functions (8). Accumulating evidence had suggested that patients with left-sided occlusion who received medical therapy had severe stroke on admission (based on NIHSS scores), and resulted in poor outcome (9, 10). However, to best of our knowledge, no significant association between lateralization of infarction and stroke outcome in patients with thrombectomy was identified. These evidences may provide an insight that left hemispheric strokes may benefit more from recanalization therapy. Moreover, both preclinical and clinical studies suggested that different neural function, nerve injury and clinical status, contributed to different treatment gains (11). In this study, we investigated whether left- or right-sided occlusions in the proximal anterior circulation differentially responded to endovascular thrombectomy in patients with comparable stroke severities.

## Methods

### Patients

Patients with proximal anterior circulation occlusion that were treated with the Solitaire stent retriever were enrolled from Yijishan Hospital of Wannan Medical College during December 2014 and August 2018. This study was approved by our local ethics committees. All enrolled patients or their surrogates provided written informed consent.

Enrolled patients met the following inclusion criteria: (1) diagnosed with acute ischemic stroke; (2) with moderate-to-severe neurologic deficits of National Institutes of Health Stroke Scale (NIHSS) scores on admission ≥5; (3) with imaging-confirmed occlusion of internal carotid artery (ICA) or M1 segment of middle cerebral artery (MCA) on computed tomography angiography (CTA), magnetic resonance angiography (MRA), or digital-subtraction angiography (DSA); (4) aged ≥18 years; (5) with a pre-stroke modified Rankin Scale (mRS) score ≤1; and (6) estimated to undergo initiation of endovascular thrombectomy within 8 h from stroke onset. We excluded patients with: (1) M2 or distal occlusion of middle cerebral artery; (2) occlusion of anterior cerebral artery; (3) posterior circulation occlusion; and (4) intracranial hemorrhage (ICH) on cranial non-contrast CT prior to treatment.

### Endovascular Thrombectomy

Thrombectomy was performed with the Solitaire stent retriever (Covidien, Irvine, CA). Perioperative management strategies were performed as previously described (12, 13). Intravenous tissue plasminogen activator (rt-PA) was allowed within 4.5 h after the onset of stroke symptoms. Direct thrombectomy without intravenous rtPA treatment was also performed in patients with heavy thrombus burdens or contraindication for intravenous rtPA. Successful recanalization was defined as a modified Thrombolysis in Cerebral Infarction (mTICI) score of 2b (50–99% reperfusion) or 3 (complete reperfusion) (14).

### Clinical and Radiologic Assessment

A series of trained neurologists performed clinical assessments including the NIHSS scores (ranging from 0 to 42) to evaluate neurologic deficits at baseline, after 24 h, at discharge or anytime indicating neurologic deterioration, and mRS scores (ranging from 0 [no symptoms] to 6 [death]) to assess global disability at baseline, after 24 h, discharge and after 90 days. The mRS at 90 days was usually assessed through a clinical interview or a telephone call if the former was not feasible.

Non-contrast CT scans were usually performed at 24 and 72 h or anytime if ICH was indicated through clinical symptoms or signs after the procedure. Symptomatic intracranial hemorrhage (SICH) included: (1) subarachnoid hemorrhage associated with clinical symptoms; (2) symptomatic intracerebral hemorrhage defined as neurologic deterioration (an increase of NIHSS score ≥4 points from baseline) and parenchymal hematoma type 2 within 36 h of thrombectomy (1, 15). Asymptomatic intracranial hemorrhage was defined as new observed ICH on cranial non-contrast CT that did not meet the diagnostic criteria of SICH. All CT, MRI and angiography were interpreted by two experienced neurologists in a blinded manner. In cases of disagreement, a third senior neurologist was consulted for a final decision.

### Statistical Analysis

The distributions of categorical variables were presented as counts (percentages) and evaluated using Chi-square or Fisher exact tests. Continuous variables with normal distributions were presented as mean (standard deviation, SD) and analyzed using Student *t*-test. Continuous variables with skewed distributions were expressed as median (interquartile range, IQR) and compared using a Mann-Whitney U test. Binary logistic regression analysis was performed to identify the predictive factors for functional outcomes. Variables with *P* < 0.1 from the univariate analysis were included for multivariate logistic regression models. A two-sided *P* < 0.05 was considered to be statistically significant. All statistical analysis was performed using SPSS software (version 23.0; IBM, Armonk, NY).

## Results

### Characteristics of Patients

Of 174 patients treated with the Solitaire stent retriever, 90 patients with left-sided occlusion and 84 patients with right-sided occlusion were enrolled. All patients had available evaluations at baseline and at 90 days. The demographic and clinical characteristics of the two groups at baseline were well balanced except for NIHSS scores on admission which were 19 (IQR, 16 to 20) in the left-sided occlusion group and 15 (IQR, 13 to 18) in the right-sided occlusion group (*P* < 0.001; Table 1).

**Table 1:** Baseline characteristics of patients with proximal anterior circulation occlusion.

**Characteristic**	**Left (*n* = 90)**	**Right (*n* = 84)**	***P***
Age, mean (SD), year	69.6 (10.2)	67.6 (12.0)	0.240
Male sex	41 (45.6)	48 (57.1)	0.127
Admission NIHSS score, median (IQR)	19 (16–20)	15 (13–18)	< 0.001
Hypertension	66 (73.3)	57 (67.9)	0.428
Diabetes mellitus	14 (15.6)	10 (11.9)	0.485
Atrial fibrillation	60 (66.7)	46 (54.8)	0.108
Systolic BP, mean (SD), mm Hg	144 (22.7)	142 (24.2)	0.171
diastolic BP, mean (SD), mm Hg	83 (13.5)	84 (14.0)	0.784
Glucose level, median (IQR), mmol/L	6.4 (5.1–9.1)	6.5 (5.4–8.2)	0.983
TOAST			0.885
Large artery disease	20 (22.2)	21 (25.0)	
Cardioembolic	61 (67.8)	54 (64.3)	
Other etiology	9 (10.0%)	9 (10.7)	
ASPECTS, median (IQR)	8 (8–10)	8 (8–9)	0.659
Site of occlusion, most proximal			0.250
Intracranial ICA	33 (36.7)	38 (45.2)	
MCA-M1	57 (63.3)	46 (54.8)	
IV-rtPA, no. (%)	13 (14.4)	5 (6.0)	0.066
Good collaterals, no. (%)	31 (34.4)	30 (35.7)	0.861
Stroke onset to groin puncture (IQR), min	240 (200–300)	265 (210–300)	0.622
Procedural time, median (IQR), min	60 (40–90)	60 (46–90)	0.447
Time to reperfusion, median (IQR), min	325 (275–370)	330 (270–370)	0.457
Successful recanalization (mTICI), no. (%)	75 (83.3)	67 (79.8)	0.543
Symptomatic ICH, no. (%)	8 (8.9)	15 (17.9)	0.081
Asymptomatic ICH, no. (%)	12 (13.3)	11 (13.1)	0.963

*ASPECTS, the Alberta Stroke Program Early Computed Tomography Score; BP, blood pressure; Good collaterals defined as American Society of Interventional and Therapeutic Neuroradiology/Society of Interventional Radiology (ASITN/SIR) ≥ 3; ICA, internal carotid artery; ICH, intracranial hemorrhage; IQR, interquartile range; IV-rtPA, intravenous alteplase; MCA, middle cerebral artery; mTICI, modified Thrombolysis in Cerebral Infarction; NIHSS, National Institutes of Health Stroke Scale; SD, standard deviation; TOAST, Trial of Org 10 172 in acute stroke treatment*.

### Intervention Details

In the left-sided occlusion group, the time from stroke onset to groin puncture, procedural time and time from stroke onset to reperfusion, were 240 min (IQR, 200–300), 60 min (IQR, 40–90), and 325 min (IQR, 275–370), respectively. Successful recanalization at the end of the procedure was achieved in 75/90 patients (83.3%). In the right-sided occlusion group, the time from stroke onset to groin puncture, procedural time and time from stroke onset to reperfusion, were 265 min (IQR, 210–300), 60 min (IQR, 46–90), and 330 min (IQR, 270–370), respectively. Successful recanalization was achieved in 67/84 patients (79.8%). Intervention parameter was adequately matched between two groups (*P* > 0.05, Table 1).

The rates of SICH were moderately higher in right-sided occlusion group compared to the left-sided occlusion group, but these differences were not significant (17.9 vs. 8.9%; *P* = 0.081; Table 1). No significant difference in the rates of asymptomatic intracranial hemorrhage were observed between the groups (13.1 vs. 13.3%; *P* = 0.963; Table 1).

### Outcome

**Figure 1:**
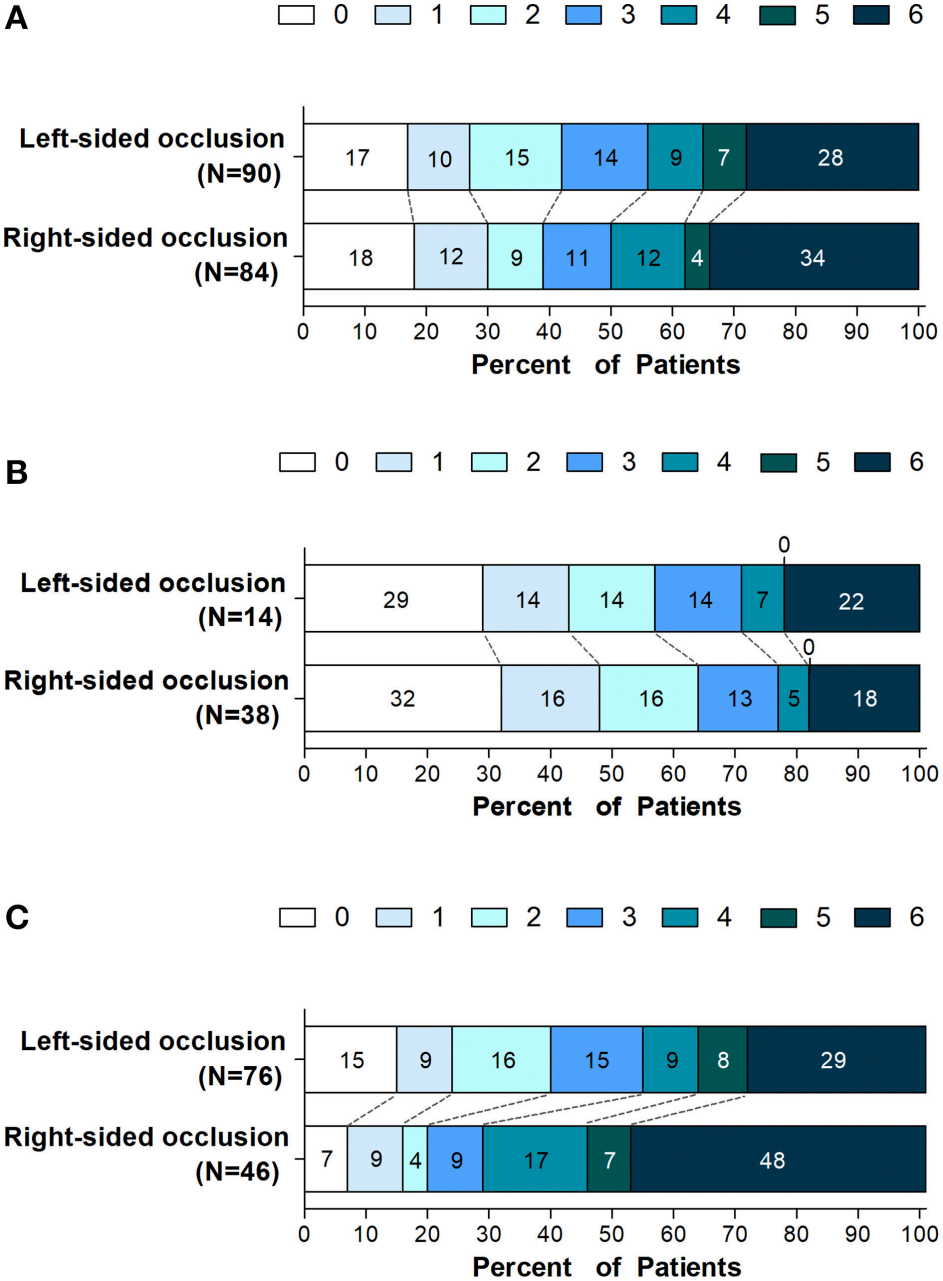
Distribution of scores on the modified rankin scale at 90 days. Shown is the distribution of 90-days modified Rankin scale ranging from 0 (no symptoms) to 6 (death) in all enrolled patients with left- or right-sided occlusion undergoing endovascular thrombectomy **(A)** and in subgroups defined according to stroke severity on admission, **(B)** patients with NIHSS score (5 to 14) and **(C)** patients with NIHSS score (≥ 15).

Left- or right-sided occlusion was not associated with functional independence or mortality in patients with endovascular thrombectomy at 90 days (Figure 1A). However, considering the imbalanced NIHSS scores at baseline and its effects on functional outcome, we performed subgroup analysis to balance the scores among moderate- (NIHSS scores, 5–14) and severe-stroke patients (NIHSS scores, 15–42) (2). The beneficial effects of thrombectomy were indicated by the mRS scores at 90 days. While the side of the occlusion was not associated with either functional independence or mortality in moderate stroke patients (Table 2 and Figure 1B), patients with the left-sided occlusion had higher rates of functional independence (39.5 vs. 19.6%; OR, 4.66; 95%CI, 1.49–14.63; *P* = 0.008) and lower rates of mortality (28.9 vs. 47.8%; OR, 0.31; 95%CI, 0.12–0.77; *P* = 0.012) in severe stroke group when adjusted for demographic and clinical characteristics. The differences were also significant when intervention parameters and complications were considered (OR, 4.23; 95%CI, 1.13–16.01; *P* = 0.032 and OR, 0.38; 95%CI, 0.14–0.98; *P* = 0.046) (Table 2, Figure 1C and Supplementary Tables 1, 2).

**Table 2:** Multivariable analysis of left or right-sided occlusion with functional outcomes.

	**Study group**		Model 1^*^		**Model 2^†^**	
**Outcome**	**Left (*N* = 90)**	**Right (*N* = 84)**	***P*-Value**	**OR (95% CI)**	***P*-Value**	**OR (95% CI)**
5 ≤ NIHSS < 15	14	38				
90-d mRS score 0–2, No. (%)	8 (57.1)	24 (63.2)	0.280	0.3 (0.03–2.67)	0.364	0.34 (0.03–3.54)
Death, No. (%)	3 (21.4)	7 (18.4)	0.959	0.95 (0.16–5.75)	0.694	1.58 (0.16–15.17)
NIHSS ≥ 15	76	46				
90-d mRS score 0–2, No. (%)	30 (39.5)	9 (19.6)	0.008	4.66 (1.49–14.63)	0.032	4.23 (1.13–16.01)
Death, No. (%)	22 (28.9)	22 (47.8)	0.012	0.31 (0.12–0.77)	0.046	0.38 (0.14–0.98)

NIHSS, National Institutes of Health Stroke Scale; mRS modified Rankin Scale.

* Model 1: Incorporated were parameters of demographic and clinical characteristics with P < 0.1 in univariate analysis (Supplementary Tables 1, 2).

† Model 2: Incorporated were parameters of Model 1+ intervention parameters and complications with P < 0.1 in univariate analysis (Supplementary Tables 1, 2).

## Discussion

In this study, we found that in patients with acute ischemic stroke due to proximal anterior circulation occlusion, patients with left-sided occlusion had significantly higher NIHSS scores on admission than those with right-sided occlusion. To reduce the heterogeneity of these scores, we divided patients into moderate and severe stroke groups. As a result, patients with left-sided occlusion who were treated with endovascular thrombectomy within 8 h after symptom onset had higher rates of functional independence and lower rates of mortality in severe stroke group, with no significant difference observed in the moderate stroke group. The results indicated that severe stroke patients with left-sided occlusion may gain more benefits from thrombectomy.

These findings confirm and extend those of previous trials. It has been suggested that patients with left hemispheric strokes had severe stroke on admission (based on NIHSS scores), leading to poorer outcomes (9, 10). The post-stroke recovery between right- and left-sided infarcts did not significantly differ (10). Di Legge et al. (16) investigated the association between the sides of the affected hemisphere and stroke outcome after intravenous rt-PA. They concluded that patients with left hemispheric strokes had higher pretreatment NIHSS scores. However, compared to patients with left hemispheric strokes, the likelihood of functional independence at 90 days of rt-PA treatment increased 2-fold, suggesting that left hemispheric stroke patients benefit more from recanalization therapy.

The left (dominant) hemisphere is considered more important than the right hemisphere in terms of functionality. The symptoms attributable to left hemispheric cerebral ischemia are more clinically obvious, while right hemispheric stroke are often underestimated (17). The NIHSS provides a valid and reproducible scale for the assessment of neurological deficits, in which 7/42 points are directly related to language related functions (typically a function of left hemisphere) and only 2/42 points to neglect (typically a function of the right hemisphere) (18). NIHSS scores thus underestimate the severity of non-dominant hemispheric infarctions in clinical trials (19). This was confirmed in our patient cohort, in which patients with left-sided occlusions had higher NIHSS scores on admission.

NIHSS scores are powerful predictors for functional outcomes (20), and have been well balanced during the process of RCTs to assess the efficiency of endovascular treatment (2–7). To explore the contribution of side differences to treatment gains observed following endovascular thrombectomy, we performed subgroup analysis according to the stroke severity (based on NIHSS scores). Given the comparable NIHSS scores, right hemispheric strokes had been reported to have a larger infarct volume than the left hemispheric strokes, and involved more motor deficits (21), on which the mRS scores mainly focus. Moreover, delayed recognition of symptoms caused by right hemispheric strokes may result in a lower reported onset time (17), which was associated with increased levels of 90-days disability for patients in whom successful reperfusion was achieved (22). More motor deficits and delayed recognition of symptoms in right hemispheric strokes may confer possible explanation to less treatment benefit of endovascular thrombectomy (indicated by the mRS scores at 90 days).

In this study, a larger number of patients received direct thrombectomy without intravenous rtPA treatment (89.6%) than those of previous studies, as all patients had been confirmed with proximal large vessel occlusions, which are predicted to occur at significantly lower rates of thrombolytic recanalization (23). The median time of stroke onset to groin puncture, and time to reperfusion were similar to the REVASCAT trial (250 vs. 269 min and 330 vs. 355 min, respectively) (5). The rates of successful recanalization (81.6%) and functional independence (40.8%) were among the ranges reported in five RCTs in 2015 (59–88 and 32.6–71%, respectively) (1–5). The rates of SICH (13.2%) and mortality (31.0%) were higher than previous RCTs (0–7.7 and 9–21%, respectively) (1–5), but were similar to another multicenter registry program from China (ACTUAL) with SICH of 16.0% and mortality of 26.3% (12). The high rates of SICH may result from the heterogeneity of enrolled patients and the diversity of the evaluated methods of SICH in different studies. In the present study, a subset of patients with ASPECTS (the Alberta Stroke Program Early Computed Tomography Score) (24) < 6 and time from stroke onset to groin puncture >6 h were enrolled. In addition, as a single center of the ACTUAL program, our study was based on the real-world practice in China, which might be another reason accounting for the differences.

There were some limitations to our study. Most importantly, this was a retrospective design which unavoidably generated selection bias that limited the generalizability of data. Although the major risk factors of functional outcomes were considered, the influence of residual and/or unknown confounding factors cannot be discounted. Secondly, this study analyzed patients from a single comprehensive stroke center with a relatively small sample size. However, all endovascular procedures were performed by a single intervention team, improving data comparability. It is therefore likely that our results reflect those of real-world practices and will be reproducible in future studies.

Our findings have implications for clinical practice. The clinical symptoms of left-sided occlusion of proximal anterior circulation may be more serious on admission. However, severe stroke patients (NIHSS ≥ 15) with left-sided large vessel occlusion may benefit more from thrombectomy within 8 h of symptom onset, which should be considered in decision-making protocols. Moreover, the side of occlusion should be considered as a predictor when designing clinical trials using NIHSS scores as baseline. These clinical implications require further verification in prospective multicenter studies using larger sample sizes.

## Data Availability

All data can be available upon request from the corresponding author.

## Author Contributions

ZZ and XH performed study design and edited the manuscript. ZL and ZC analyzed data and prepared the manuscript. SZ, LM, and QY collected clinical data and interpreted the images. All authors approved the final manuscript.

### Conflict of Interest Statement

The authors declare that the research was conducted in the absence of any commercial or financial relationships that could be construed as a potential conflict of interest.
