# Supplementary material for: Severe Stroke Patients With Left-Sided Occlusion of the Proximal Anterior Circulation Benefit More From Thrombectomy
Source: Front Neurol. 2019 May 28;10:551. doi: 10.3389/fneur.2019.00551 (PMC6546891; doi:10.3389/fneur.2019.00551)
Supplement: Supplementary file 1 [file Table_1.DOCX]

Supplementary Material

Supplementary Table 1: Univariate Analysis for Functional Independence at 90 Days According to the Stroke Severity

|  | NIHSS<15 | | | NIHSS≥15 | | |
| --- | --- | --- | --- | --- | --- | --- |
| Variables | Good outcome  N=32 | Poor outcome  N=20 | *P* | Good outcome  n=39 | Poor outcome  N=83 | *P* |
| Age, mean (SD), y | 63.9(12.8) | 71.4(11.1) | 0.037 | 64.9(10.6) | 71.6(9.5) | 0.001 |
| Male sex | 19(59.4) | 13(63.0) | 0.685 | 21(53.8) | 36(43.4) | 0.281 |
| Admission NIHSS score, median (IQR) | 12(10-14) | 13(12-14) | 0.029 | 19(17-20) | 19(16-20) | 0.483 |
| Hypertension | 16(50.0) | 16(80.0) | 0.036 | 31(79.5) | 60(72.3) | 0.396 |
| Diabetes mellitus | 2(6.3) | 3(15%) | 0.312 | 5(12.8) | 14(16.9) | 0.567 |
| Atrial fibrillation | 14(43.8) | 12(60.0) | 0.257 | 19(48.7) | 61(73.5) | 0.008 |
| Systolic BP, mean (SD), mm Hg | 131(24.8) | 144(22.4) | 0.065 | 144(20.1) | 148(23.2) | 0.346 |
| diastolic BP, mean (SD), mm Hg | 81(17.7) | 82(10.3) | 0.742 | 85(11.0) | 84(14.0) | 0.737 |
| Glucose level, median (IQR), mmol/L | 5.6(4.6-6.5) | 7.5(5.9-8.7) | 0.035 | 5.4(4.8-6.8) | 7.2(5.8-10.0) | 0.007 |
| TOAST |  |  | 0.574 |  |  | 0.005 |
| Large artery disease | 10(31.3) | 5(25.0) |  | 11(28.2) | 15(18.1) |  |
| Cardioembolic | 15(46.9) | 15(75.0) |  | 20(51.3) | 65(78.3) |  |
| Other etiology | 7(21.9) | 0(0.0) |  | 8(20.5) | 3(3.6) |  |
| ASPECTS>7, no. (%) | 29(90.6) | 14(70.0) | 0.044 | 39(100.0) | 59(71.1) | <0.001 |
| Site of occlusion, most proximal |  |  | 0.512 |  |  | 0.033 |
| Intracranial ICA | 7(21.9) | 6(30.0) |  | 13(33.3) | 45(54.2) |  |
| MCA-M1 | 25(78.1) | 14(70.0) |  | 26(66.7) | 38(45.8) |  |
| Left-sided occlusion | 8(25.0) | 6(70.0) | 0.693 | 30(76.9) | 46(55.4) | 0.025 |
| IV-rtPA, no. (%) | 6(18.8) | 1(5.0) | 0.188 | 3(7.7) | 8(9.6) | 0.727 |
| Good collaterals, no. (%) | 22(68.8) | 5(25.0) | 0.003 | 17(43.6) | 17(20.5) | 0.009 |
| Stroke onset to groin puncture (IQR), min | 270(213-317) | 255(185-300) | 0.433 | 260(225-300) | 240(200-300) | 0.362 |
| Procedural time, median (IQR), min | 55(35.5-87.5) | 73(50-120) | 0.022 | 60(40-60) | 70(48-100) | 0.029 |
| Time to reperfusion, median (IQR), min | 330(266-389) | 340(290-378) | 0.963 | 320(270-370) | 330(275-370) | 0.758 |
| Successful recanalization (TICI), no. (%) | 28(87.5) | 15(75.0) | 0.254 | 36(92.3) | 63(75.9) | 0.041 |
| Symptomatic ICH, no. (%) | 1(3.1) | 5(25.0) | 0.040 | 1(2.6) | 16(19.3) | 0.036 |
| Asymptomatic ICH, no. (%) | 0 (0.0) | 2(10.0) | 0.143 | 6(15.4) | 15(18.1) | 0.714 |

ASPECTS the Alberta Stroke Program Early Computed Tomography Score; BP blood pressure; Good collaterals defined as American Society of Interventional and Therapeutic Neuroradiology/Society of Interventional Radiology (ASITN/SIR) ≥ 3; ICA internal carotid artery; ICH intracranial hemorrhage; IQR interquartile range; IV-rtPA intravenous alteplase; MCA middle cerebral artery; mTICI modified Thrombolysis in Cerebral Infarction; NIHSS National Institutes of Health Stroke Scale; SD standard deviation; TOAST Trial of Org 10 172 in acute stroke treatment

Supplementary Table 2：Univariate Analysis for Mortality at 90 Days According to the Stroke Severity

|  | NIHSS<15 | | | NIHSS≥15 | | |
| --- | --- | --- | --- | --- | --- | --- |
| Variables | Live  N=42 | Death  N=10 | *P* | Live  N=78 | Death  N=44 | *P* |
| Age, mean (SD), y | 65(12.7) | 72.9(10.7) | 0.097 | 68.1(10.4) | 71.9(9.8) | 0.054 |
| Male sex | 25(59.5) | 7(70.0) | 0.543 | 36(46.2) | 21(47.7) | 0.867 |
| Admission NIHSS score, median (IQR) | 12(10-14) | 13(11-14) | 0.239 | 19(17-10) | 18(16-20) | 0.930 |
| Hypertension | 24(57.1) | 8(80.0) | 0.196 | 44(48.4) | 47(51.6) | 0.172 |
| Diabetes mellitus | 4(9.5) | 1(10.0) | 0.963 | 12(15.4) | 7(15.9) | 0.939 |
| Atrial fibrillation | 18(42.9) | 8(80.0) | 0.049 | 48(61.5) | 32(72.7) | 0.214 |
| Systolic BP, mean (SD), mm Hg | 134(24.1) | 143(26.2) | 0.267 | 144(21.1) | 150(24.0) | 0.182 |
| diastolic BP, mean (SD), mm Hg | 81(16.2) | 83(10.5) | 0.848 | 84.9(12.1) | 83.7(14.7) | 0,603 |
| Glucose level, median (IQR), mmol/L | 5.9(4.7-7.3) | 8.1(6.7-9.7) | 0.102 | 6.0(5.1-7.4) | 8.3(6.2-11.0) | 0.002 |
| TOAST |  |  | 0.271 |  |  | 0.097 |
| Large artery disease | 14(33.3) | 1(10.0) |  | 20(25.6) | 6(13.6) |  |
| Cardioembolic | 21(50.0) | 9(90.0) |  | 49(62.8) | 36(81.8) |  |
| Other etiology | 7(16.7) | 0(0.0) |  | 9(11.5) | 2(4.5) |  |
| ASPECTS>7, no. (%) | 38(90.5) | 5(50.0) | 0.006 | 67(85.9) | 31(70.5) | 0.043 |
| Site of occlusion, most proximal |  |  | 0.685 |  |  | 0.125 |
| Intracranial ICA | 10(23.8) | 3(30.0) |  | 33(42.3) | 25(56.8) |  |
| MCA-M1 | 32(76.2) | 7(7.0) |  | 45(57.7) | 19(43.2) |  |
| Left-sided occlusion | 11(26.2) | 3(30.0) | 0.807 | 54(69.2) | 22(50.0) | 0.037 |
| IV-rtPA, no. (%) | 6(14.3) | 1(10.0) | 0.723 | 8(10.3) | 3(6.8) | 0.527 |
| Good collaterals, no. (%) | 24(57.1) | 3(30.0) | 0.134 | 29(37.2) | 5(11.4) | 0.004 |
| Stroke onset to groin puncture (IQR), min | 260(100-302) | 285(225-323) | 0.675 | 265(214-300) | 240 (187-300) | 0.187 |
| Procedural time, median (IQR), h | 60(40-93) | 63(40-98) | 0.443 | 60(40-90) | 78(43-107) | 0.166 |
| Time to reperfusion, median (IQR), h | 330(269-383) | 343(288-383) | 0.746 | 330(279-371) | 315(263-368) | 0.585 |
| Successful recanalization (TICI), no. (%) | 34(81.0) | 9(90.0) | 0.505 | 67(85.9) | 32(72.7) | 0.079 |
| Symptomatic ICH, no. (%) | 1(2.4) | 5(50.0) | 0.002 | 7(9.0) | 10(22.7) | 0.041 |
| Asymptomatic ICH, no. (%) | 1(2.4) | 1(10.0) | 0.351 | 14(17.9) | 7(15.9) | 0.774 |

ASPECTS the Alberta Stroke Program Early Computed Tomography Score; BP blood pressure; Good collaterals defined as American Society of Interventional and Therapeutic Neuroradiology/Society of Interventional Radiology (ASITN/SIR) ≥ 3; ICA internal carotid artery; ICH intracranial hemorrhage; IQR interquartile range; IV-rtPA intravenous alteplase; MCA middle cerebral artery; mTICI modified Thrombolysis in Cerebral Infarction; NIHSS National Institutes of Health Stroke Scale; SD standard deviation; TOAST Trial of Org 10 172 in acute stroke treatment
